# Dual-energy contrast-enhanced digital mammography: initial clinical results of a multireader, multicase study

**DOI:** 10.1186/bcr3210

**Published:** 2012-06-14

**Authors:** Clarisse Dromain, Fabienne Thibault, Felix Diekmann, Eva M Fallenberg, Roberta A Jong, Marcia Koomen, R Edward Hendrick, Anne Tardivon, Alicia Toledano

**Affiliations:** 1 Department of Radiology, Institut de cancérologie Gustave-Roussy, 39 rue Camille Desmoulin, Villejuif, 94805 France; 2 Department of Radiology, Institut Curie, 26 rue d'Ulm, Paris, 75005, France; 3 Department of Radiology, University Hospital Charite, 1 charitéplatz, D - 10117 Berlin, Germany; 4 Department of Medical Imaging, Sunnybrook and Women's College Health Sciences Center, 2075 Bayview Avenue, MG178 Toronto, ON, Canada M4N 3M5; 5 Department of Radiology, University of North Carolina at Chapel Hill, Chapel Hill, NC,27599-7510 USA; 6 Department of Radiology, University of Colorado, Denver, Aurora, CO 80045, USA; 7 Statistic, Statistics Collaborative, 1625 Massachusetts Avenue, NW, Suite 600, Washington, DC 20036, USA

## Abstract

**Introduction:**

The purpose of this study was to compare the diagnostic accuracy of dual-energy contrast-enhanced digital mammography (CEDM) as an adjunct to mammography (MX) ± ultrasonography (US) with the diagnostic accuracy of MX ± US alone.

**Methods:**

One hundred ten consenting women with 148 breast lesions (84 malignant, 64 benign) underwent two-view dual-energy CEDM in addition to MX and US using a specially modified digital mammography system (Senographe DS, GE Healthcare). Reference standard was histology for 138 lesions and follow-up for 12 lesions. Six radiologists from 4 institutions interpreted the images using high-resolution softcopy workstations. Confidence of presence (5-point scale), probability of cancer (7-point scale), and BI-RADS scores were evaluated for each finding. Sensitivity, specificity and ROC curve areas were estimated for each reader and overall. Visibility of findings on MX ± CEDM and MX ± US was evaluated with a Likert scale.

**Results:**

The average per-lesion sensitivity across all readers was significantly higher for MX ± US ± CEDM than for MX ± US (0.78 vs. 0.71 using BIRADS, p = 0.006). All readers improved their clinical performance and the average area under the ROC curve was significantly superior for MX ± US ± CEDM than for MX ± US ((0.87 vs 0.83, p = 0.045). Finding visibility was similar or better on MX ± CEDM than MX ± US in 80% of cases.

**Conclusions:**

Dual-energy contrast-enhanced digital mammography as an adjunct to MX ± US improves diagnostic accuracy compared to MX ± US alone. Addition of iodinated contrast agent to MX facilitates the visualization of breast lesions.

## Introduction

Though widely established as the only screening imaging modality that can reduce breast cancer mortality, mammography (MX) has some limitations, such as lesions masked by normal fibroglandular tissue, lesions seen on only one view, and subtle architectural distortions [1,2]. Partly because of these limitations, MX misses about 20% of invasive breast cancers [3,4]. Full-field digital mammography (FFDM) enables high-quality breast images with higher-contrast resolution, improved dynamic range, and rapid processing of data and images compared with screen film MX. FFDM has been shown to provide increased accuracy in screening pre- or peri-menopausal women, women younger than 50, and women with dense breasts [5]. Moreover, FFDM offers the possibility of developing new and advanced applications for breast imaging. Contrast-enhanced digital mammography (CEDM) with injection of an iodinated contrast agent is one of them.

Contrast agent has been used for many years in both computed tomography (CT) and magnetic resonance imaging (MRI) examinations to explore angiogenesis in breast carcinoma by tracking the uptake and washout of contrast agent in tissues. Iodinated contrast-enhanced conventional CT was shown to be useful for detecting breast carcinoma [6]. However, conventional CT results in a high-radiation dose to the breast and chest wall. Recent studies on dedicated breast CT with radiation doses similar to or slightly higher than those of two-view MX have shown that malignant lesions were significantly more conspicuous at contrast-enhanced breast CT than at MX and have suggested the potential usefulness of quantitating enhancement of breast lesions in predicting malignancy [6,7]. Breast MRI using gadolinium-based contrast agents is currently considered the most sensitive imaging technique for the detection of breast carcinoma, and multiple indications have been established for breast MRI [8]. However, breast MRI has a variable specificity and positive predictive value and is more time-consuming and approximately 10 times more expensive than MX [9,10].

Investigational clinical results on CEDM have been published during the last few years, suggesting that the technique may be a useful adjunct to MX with lesion contrast uptake information [11-14]. Two CEDM examination techniques have been investigated: temporal subtraction and dual-energy. The study by Lewin and colleagues [11] is the only published preliminary clinical experience using dual-energy CEDM. The authors showed the technical and clinical feasibility of this technique and reported a sensitivity of 92% and a specificity of 83% for the detection of breast carcinoma [11]. This study was limited, however, in that the x-ray beam was not optimized for dual-energy acquisition and the number of subjects was small. Moreover, no comparison was made between CEDM and MX interpreted in association with ultrasonography (US), a routine of care in the diagnostic setting.

### Purpose

The objective of the multireader study presented in this article was to quantify the diagnostic accuracy of dual-energy CEDM as an adjunct to MX ± US, with that of MX ± US, in a larger patient cohort. We also evaluated lesion visibility with MX ± CEDM compared with MX ± US.

## Materials and methods

### Patients

From March 2007 to March 2008, 122 consecutive patients provided written informed consent and participated in the study. The study was approved by the ethics committee (Comité de protection des personnes, Ile de France) and the institutional review board. Inclusion criteria were recalls from screening with unresolved findings after MX and ultrasound. Exclusion criteria were isolated clusters of microcalcifications, pregnancy or possible pregnancy, or a history of allergic reaction to an iodinated contrast agent. Data from two patients were excluded from analysis because these patients were later determined to be ineligible (one patient had breast implants and one patient was undergoing chemotherapy at the time of imaging). Data from the first 10 patients were used for training cases and were also excluded from the analysis. The remaining 110 patients, who had a mean age ± standard deviation (SD) of 57 ± 11.8 years, formed the study group. Reference standard for each lesion was obtained through surgery (82 lesions; 55%), core biopsy (42 lesions; 28%), fine-needle aspiration (11 lesions; 7%), or follow-up of at least 3 years, median 47 months (12 findings; 8%, all benign); one lesion with unknown reference standard procedure was classified as benign (Table 1). The same image data set used in this study was used in an investigational single-reader study that evaluated the clinical performance of CEDM in comparison with MX and MX ± US [15]. This paper reports the results of a multireader study evaluating the diagnostic value of CEDM as an adjunct to MX ± US (CEDM ± MX ± US versus MX ± US).

**Table 1:** Characteristics of study lesions

	Benign (*n *= 64)	Malignant (*n *= 84)	Total (*n *= 148)
Palpable			
Yes	14 (23)	28 (34)	42 (29)
No	48 (77)	55 (66)	103 (71)
Laterality			
Right	41 (64)	48 (57)	89 (60)
Left	23 (36)	36 (43)	59 (40)
Type of findings on MX			
Mass	27 (42)	46 (55)	73 (49)
Asymmetry	9 (14)	4 (5)	13 (9)
Distortion	3 (5)	3 (4)	6 (4)
Intramammary node	1 (2)	0 (0)	1 (1)
Mass ± calcs	6 (9)	20 (24)	26 (18)
Asymmetry ± calcs	1 (2)	0 (0)	1 (1)
Calcs	3 (5)	8 (10)	11 (7)
Unknown	14 (22)	3 (4)	17 (11)
Procedure			
Surgery	24 (38)	58 (69)	82 (55)
Core biopsy	19 (30)	23 (27)	42 (28)
FNAB	8 (12)	3 (4)	11 (7)
Follow-up	13 (20)	0 (0)	13 (9)
Histological grade			
G1		26 (31)	
G2		33 (39)	
G3		12 (14)	
Unknown		13 (15)	
US images available			
Yes	51 (80)	73 (87)	124 (84)
No	13 (20)	11 (13)	24 (16)

Values are presented as number and percentage unless otherwise noted. calcs, calcifications; FNAB, fine-needle aspiration biopsy; MX, mammography; US, ultrasonography.

### Dual-energy CEDM examinations

All CEDM examinations were performed with an experimental device that was developed by GE Healthcare (Chalfont St. Giles, UK) and that allowed dual-energy CEDM acquisitions. The system used was a commercially available FFDM system (Senographe DS; GE Healthcare) that was modified to shape the x-ray spectrum specifically for CEDM [16]. Dual-energy CEDM was performed by acquiring a pair of low- and high-energy images in quick succession during a single breast compression. Low-energy images were acquired with molybdenum (Mo) or rhodium (Rh) target and Mo or Rh filter at peak kilovoltage (kVp) values ranging from 26 to 31, ensuring that the entire x-ray spectrum was below the k-edge of iodine (33.2 keV). High-energy images were acquired with Mo target and a double-layer filter (0.3 mm copper ± 0.3 mm aluminum) at 45 to 49 kVp, ensuring that the average energy of the x-ray spectrum was just above the k-edge of iodine. Exposures were performed by using an anti-scatter grid. Iodine-enhanced images were generated from the low- and high-energy images. They display the regions of iodine contrast uptake while canceling non-enhancing anatomic noise in the images. The appropriate combination of low- and high-energy images is determined on the basis of simulations of low- and high-energy signal levels and depends on low- and high-energy spectra as well as the compressed breast thickness [17].

A catheter was inserted into the antecubital vein of the arm contralateral to the breast of concern. A one-shot intravenous injection of 1.5 mL/body weight of non-ionic contrast media (Xenetix 300; Guerbet, Villepinte, France) was then performed by the radiographer in charge by using a power injector (Medrad, Pittsburgh, PA, USA). The injection rate was 3 mL/s. Two minutes after the initiation of contrast administration, the breast was compressed in the mediolateral oblique (MLO) view, and a low- and high-energy pair of images was acquired within 20 seconds of one another. The breast was then compressed in the craniocaudal (CC) position, and a new low- and high-energy pair of exposures was acquired 4 minutes after the initiation of contrast administration. A combination of low- and high-energy images was performed through appropriate image processing to generate two iodine-enhanced images with contrast uptake information, one in MLO and the other in CC projection.

**Figure 1:**
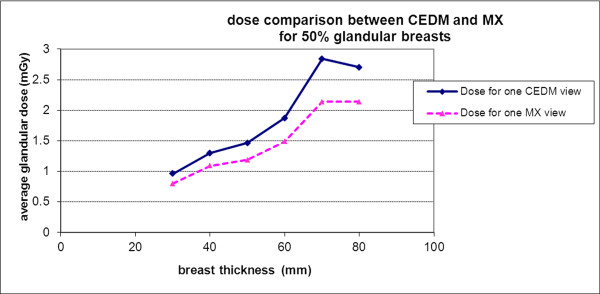
**Estimated average glandular dose per view (in milligrays) for contrast-enhanced digital mammography (CEDM) in comparison with mammography (MX) for 50% glandular breast**. The solid line is the dose for one CEDM view, and the dotted line is for one MX view.

The total x-ray dose per view delivered to the patient from the pair of low- and high-energy images was between 0.7 and 3.6 mGy, depending on breast thickness (30 to 80 mm) and tissue composition (0% to 100% glandular tissue) (Figure 1). A model of the imaging chain was used to estimate the average glandular dose. It is based on the bremsstrahlung spectrum model by Birch and Marshall [18], and characteristic rays were added from tabulated data. Scattered radiation is included in the model: it is expressed as a fraction of the primary radiation, whose value depends on the breast thickness and has been determined experimentally. Estimated values of the dose for standard MX spectra were in good agreement with published data from Wu and colleagues [19], and the mean difference was 3%. For the high-energy spectra, the comparison of average glandular dose for an Mo/Cu 49 kVp spectrum gave approximately 5% relative difference compared with Boone's results [20]. The dose estimated from low- and high-energy views combined is about 1.2 times the dose delivered in standard single-view digital MX.

### Image analysis

Seven experienced breast radiologists from five institutions read MX, US, and CEDM images independently and were blinded to each patient's cancer status. MX and US examinations used for the reading session were those performed by the referring physician in charge as part of standard of care before inclusion in the study. Readings were performed on individual workstations, loaded with all cases, and calibrated within controlled ambient lighting conditions. Prior to study readings, a training session was run by a radiologist who participated in case collection. Ten training cases were provided to familiarize radiologists with CEDM and the reading protocol. Iodine-enhanced CEDM images were reviewed by using reading criteria based on contrast enhancement intensity and morphology similar to those described in the Breast Imaging, Reporting and Data System (BI-RADS) MRI lexicon developed by the American College of Radiology [8]. For MX examination only, the two CC and MLO images were reviewed without any additional views. For US examinations only, still images (screen saved by the referring radiologist who performed the free-hand US examination) were reviewed. Training cases, as well as readings by the radiologist who was from the collecting institution and who performed the single-reader study previously published, were excluded from the analysis. Since the goal of our study was to evaluate the clinical benefit of CEDM as an adjunct to MX ± US, each radiologist interpreted each case independently and in two sequential steps during the same reading session: (a) MX ± US and (b) MX ± US ± CEDM. At each step, readers were required to assess the case, localize the findings, give BI-RADS scores, then complete and save an electronic data form. To conclude the assessment of a case, the truth (location and nature of known lesions) was communicated after electronic data submission and each reader was asked to rate relative feature visibility with MX ± CEDM and MX ± US displayed side by side.

Data forms were completed with the following information: patient identification, breast density on MX (BI-RADS scores of 1 to 4), location of each finding (limited to the three most suspicious per breast), type of each finding (asymmetry, calcification, mass/density, calcification ± mass, and scar/distortion), confidence in presence of each finding (5-step scale in which 1 = very low and 5 = very high), probability of malignancy for each finding (7-step scale in which 1 = extremely low and 7 = extremely high), BI-RADS classification (1 to 5, BI-RADS scores of 0 and 6 were not allowed), and side-by-side feature visibility rating with MX ± CEDM versus MX ± US (5-step Likert scale, -2 = MX ± US definitely superior and ± 2 = MX ± CEDM definitely superior).

### Statistical analysis methods

The primary end point was area under the receiver operating characteristic (ROC) curve. A case was classified as negative if the patient had no malignant breast lesions by the reference standard or as positive if she had at least one proven malignant breast lesion. For each reader in each reading paradigm, we obtained the per-patient BI-RADS score as the maximum of the 5-point BI-RADS scores assigned by the reader to all findings in that patient; the per-patient 7-point probability of cancer score was obtained similarly. Smooth ROC curves were estimated on the basis of each of the BI-RADS and probability of cancer scales by using a proper binormal model [21,22] in DBM MRMC software from the University of Chicago [23]. Per-patient analysis of accuracy included the secondary end points of sensitivity and specificity, and a positive test was defined as a score of 4 or higher for both BI-RADS and probability of cancer scales. The achieved statistical power of ROC MRMC was greater than 80%.

Additional secondary end points were lesion-based sensitivity and false-positive marks per negative case. A reader was determined to have successfully located a real lesion (malignant or benign) if he or she marked a finding in a quadrant matching the true location of the lesion and described a finding type consistent with the true finding type. When finding type could not be determined for truth, any finding type in the appropriate location was considered a match. When neither finding type nor location was available from truth, any marked finding was considered to match the real lesion. A reader's mark was considered a true positive if it matched a malignant lesion and the reader's score for that finding was 4 or higher for each of the BI-RADS and probability of cancer scales. A reader's mark was considered a false positive if it matched a benign lesion or did not match a real lesion and if the reader's score for that finding was 4 or higher. For each reader in each reading paradigm, sensitivity to malignant lesions was computed as the number of true-positive marks divided by the number of malignant lesions. This result was complemented by computing the average number of false-positive marks per case in cases with no malignant lesions.

Feature visibility ratings were tabulated for each reader and summarized across all readers. A confidence interval (CI) for the proportion of MX ± CEDM ratings as 'similar', 'slightly better', or 'better' was obtained by using the model of Obuchowski and Rockette (1995) [24], considering the side-by-side comparison to be a single test condition.

Multireader, multicase ROC curve analysis used the method of Dorfman, Berbaum, and Metz (1992) [25] as implemented in DBM MRMC version 2.2 [26-31]. Within-reader comparisons of sensitivity and specificity used McNemar's chi-squared test, and comparison of false-positive marks per case used the Wilcoxon signed rank test. Multireader, multicase analysis of per-patient sensitivity and specificity and of per-lesion sensitivity and false-positive marks per case used the models of Obuchowski and Rockette (1995) [24] and was implemented in Splus (version 6.2 for Windows, Professional Edition). Statistical tests were performed at a significance level alpha of 0.05. Two-sided 95% CIs were used to quantify uncertainty.

## Results

The 110 eligible patients presented with a total of 148 breast lesions: 82 patients (75%) had a single breast lesion, 18 (16%) had two, and 10 (9%) had three. Eighty-four lesions were malignant and 64 were benign. Most lesions (103, or 70%) were non-palpable. The mean histological size of lesions was 15.9 mm (SD = 10.9 mm, *n *= 70). Lesions generally appeared on MX as masses or similar (93, or 63%: 73 masses, 13 asymmetries, six distortions, and one intramammary node) and less often as masses with calcifications (27, or 18%, including one asymmetry with calcifications) or isolated clusters of calcifications (11, or 7%). Seventeen lesions (11%) were not visible on MX alone, even retrospectively. Ultrasound still images ('screen saves') were available for 90 patients (82%); for the remaining 20 (18%), no lesions were seen on US and therefore no still images were obtained. Only one mild adverse reaction to the administration of iodinated contrast agent - a limited urticaria that did not require specific treatment - was observed. The patient was observed for 20 minutes to ensure clinical stability and recovery.

**Figure 2:**
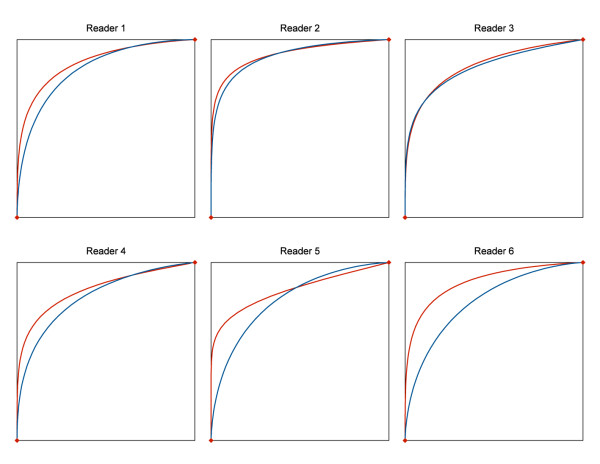
**Estimated proper binormal receiver operating characteristic curves from BI-RADS assessments for the six readers**. The red line is MX ± US ± CEDM, and the blue line is MX ± US. BI-RADS, Breast Imaging, Reporting and Data System; CEDM, contrast-enhanced digital mammography; MX, mammography; US, ultrasonography.

**Figure 3:**
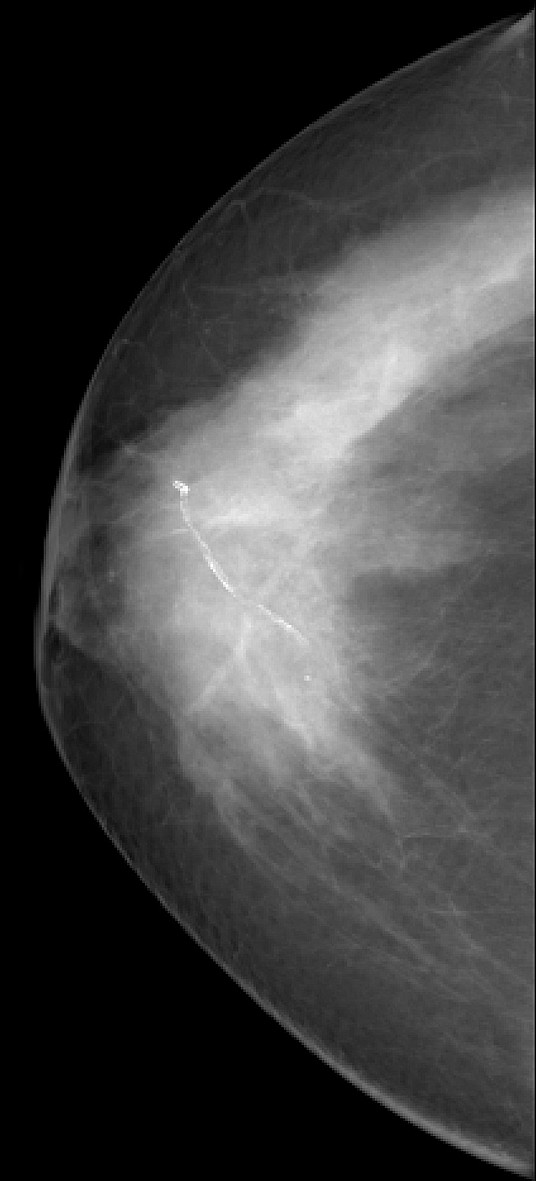
**Invasive lobular carcinoma in a 67-year-old woman with left nipple stiffness and retraction**. The right craniocaudal mammogram is normal.

**Figure 4:**
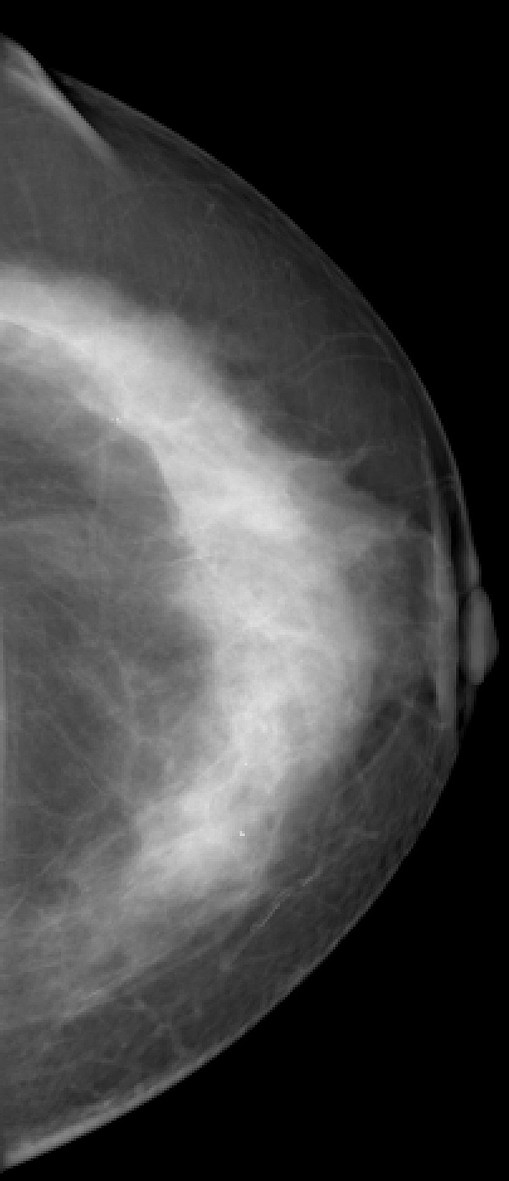
**Invasive lobular carcinoma in a 67-year-old woman with left nipple stiffness and retraction**. The left craniocaudal mammogram shows a left cutaneous nipple and areolar thickness with no obvious lesion in the breast parenchyma.

**Figure 5:**
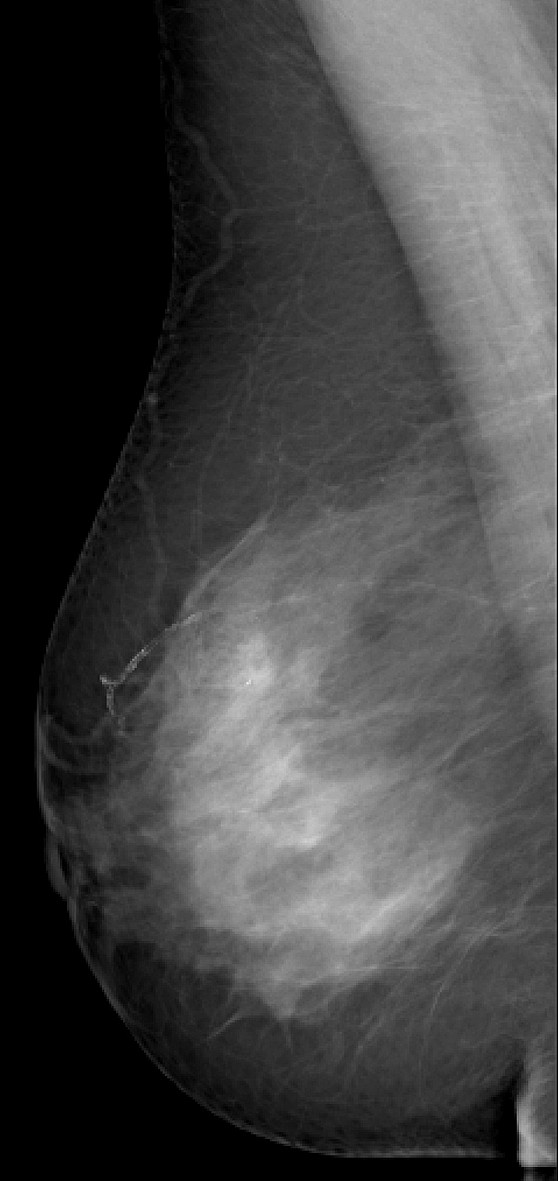
**Invasive lobular carcinoma in a 67-year-old woman with left nipple stiffness and retraction**. The right mediolateral oblique mammogram is normal.

**Figure 6:**
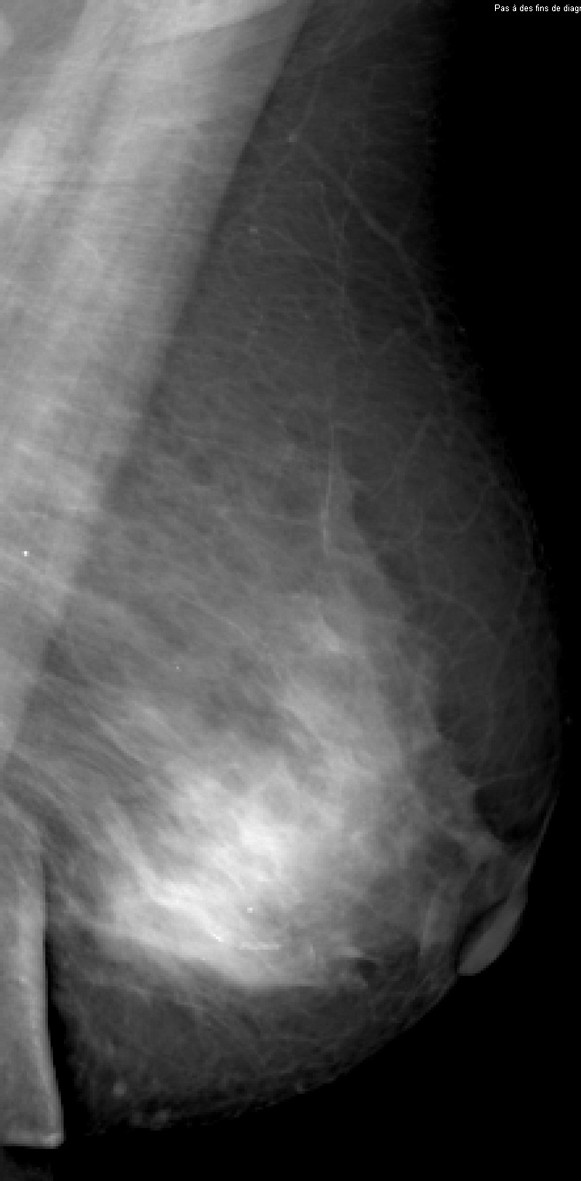
**Invasive lobular carcinoma in a 67-year-old woman with left nipple stiffness and retraction**. The left mediolateral oblique mammogram shows a left cutaneous nipple and areolar thickness with no obvious lesion in the breast parenchyma.

**Figure 7:**
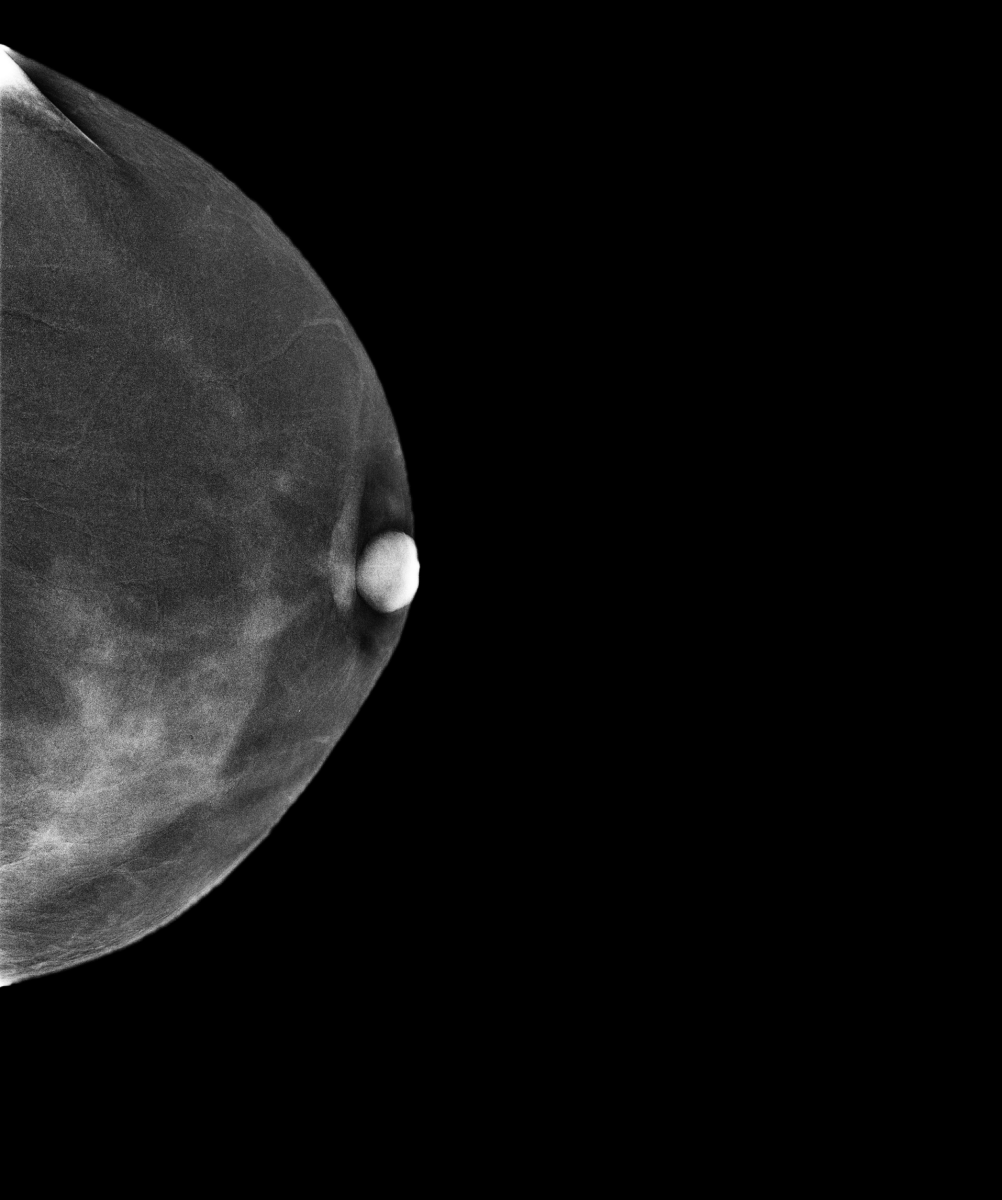
**Invasive lobular carcinoma in a 67-year-old woman with left nipple stiffness and retraction**. The iodine-enhanced, contrast-enhanced digital mammography, craniocaudal image clearly depicts non-mass regional enhancement in the inner quadrant.

**Figure 8:**
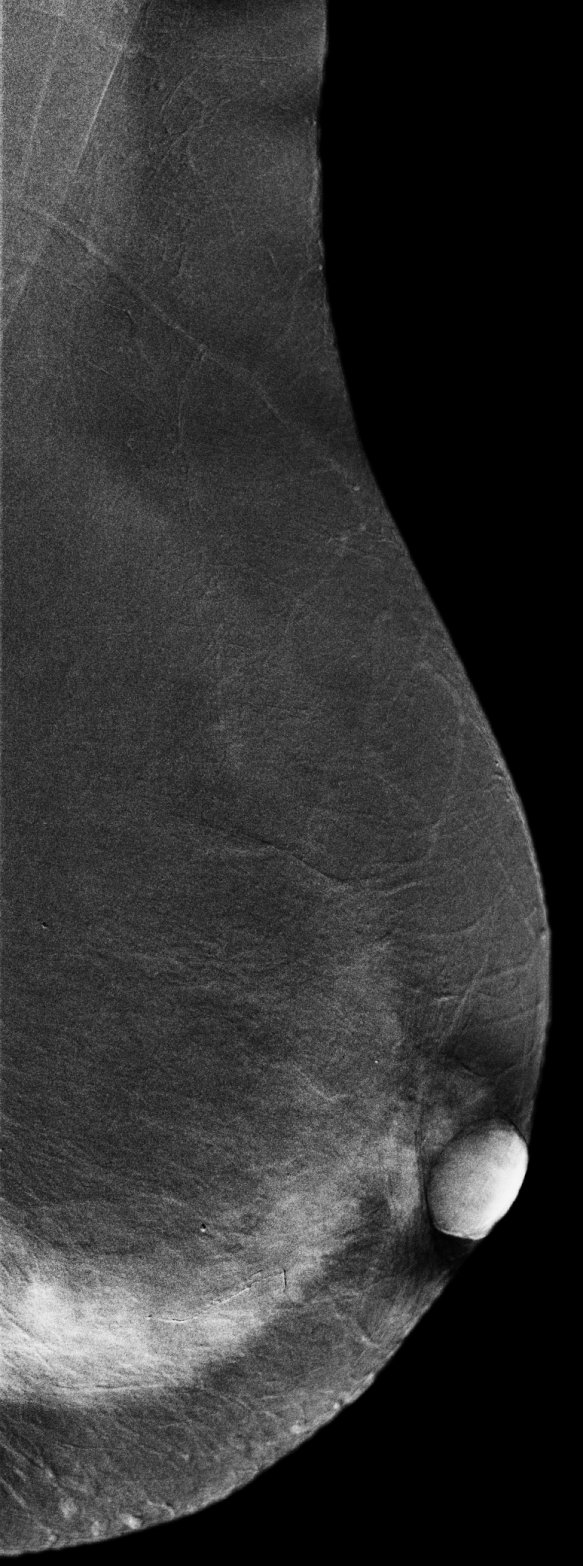
**Invasive lobular carcinoma in a 67-year-old woman with left nipple stiffness and retraction**. The iodine-enhanced, contrast-enhanced digital mammography, mediolateral oblique images clearly depicts non-mass regional enhancement in the inferior quadrant.

**Figure 9:**
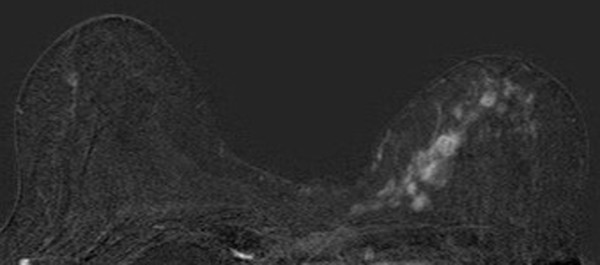
**Invasive lobular carcinoma in a 67-year-old woman with left nipple stiffness and retraction**. The contrast-enhanced transaxial breast magnetic resonance image shows the same non-mass regional enhancement than CEDM images.

Area under the ROC curve increased for each reader with the addition of CEDM (Table 2 and Figure 2). Overall multireader areas under the ROC curve were 0.827 (standard error (SE) = 0.036) for MX ± US and 0.871 (SE = 0.027) for MX ± US ± CEDM. The average increase in ROC area, 0.043 (SE = 0.019), was statistically significant (*P *= 0.045). When BI-RADS score of 4 or 5 was used to define a positive test, per-patient sensitivity increased slightly for three of six study readers and decreased slightly for the other three; the average change was -0.003: SE = 0.022, *P *= 0.910 not significant (NS) (Table 3) (Figures 3, 4, 5, 6, 7, 8, 9). Specificity increased for five of six study readers and decreased for the sixth; the average change was 0.040: SE = 0.040, *P *= 0.336 NS (Table 3). Similar results were obtained using the 7-point probability of malignancy scale.

**Table 2:** Areas under proper binormal receiver operating characteristic curves constructed from responses on the BI-RADS scale

Reader	MX ± US	MX ± US ± CEDM	Increase in Az
1	0.835 ± 0.047	0.872 ± 0.041	0.037 ± 0.026
2	0.907 ± 0.042	0.916 ± 0.030	0.009 ± 0.036
3	0.843 ± 0.042	0.851 ± 0.044	0.009 ± 0.031
4	0.809 ± 0.047	0.849 ± 0.040	0.041 ± 0.026
5	0.791 ± 0.050	0.844 ± 0.040	0.053 ± 0.041
6	0.780 ± 0.049	0.891 ± 0.032	0.111 ± 0.033 (*P *= 0.001)
Overall	0.827 ± 0.036	0.871 ± 0.027	0.043 ± 0.019
95% CI	0.756 to 0.899	0.817 to 0.92	0.001 to 0.085 (*P *= 0.045)

Az, area under receiver operating characteristic curve estimated by using proper binormal model; BI-RADS, Breast Imaging, Reporting and Data System; CEDM, contrast-enhanced digital mammography; CI, confidence interval, inference used MRMC methods; MX, mammography; US, ultrasonography.

**Table 3:** Sensitivity (per-patient) and specificity using BI-RADS score of at least 4 to define a positive test

Reader	MX ± US	MX ± US ± CEDM	Difference	*P *value
Sensitivity				
1	0.875 ± 0.042	0.922 ± 0.034	0.047	0.25
2	0.953 ± 0.027	0.938 ± 0.030	-0.016	1.00
3	0.922 ± 0.034	0.938 ± 0.030	0.016	1.00
4	0.969 ± 0.022	0.953 ± 0.027	-0.016	1.00
5	0.938 ± 0.030	0.859 ± 0.044	-0.078	0.07
6	0.875 ± 0.042	0.906 ± 0.037	0.031	0.68
Overall	0.922 ± 0.028	0.919 ± 0.031	-0.003 ± 0.022	0.91
95% CI	0.866 to 0.977	0.858 to 0.981	-0.052 to 0.047	
Specificity				
1	0.565 ± 0.074	0.457 ± 0.074	-0.109	0.18
2	0.478 ± 0.074	0.587 ± 0.073	0.109	0.23
3	0.413 ± 0.073	0.457 ± 0.074	0.043	0.68
4	0.174 ± 0.057	0.239 ± 0.064	0.065	0.37
5	0.326 ± 0.07	0.413 ± 0.073	0.087	0.34
6	0.565 ± 0.074	0.609 ± 0.073	0.043	0.77
Overall	0.42 ± 0.073	0.46 ± 0.071	0.040	0.336
95% CI	0.262 to 0.578	0.308 to 0.613	-0.046 to 0.126	

Values are presented as estimate ± standard error unless otherwise indicated. BI-RADS, Breast Imaging, Reporting and Data System; CEDM, contrast-enhanced digital mammography; CI, confidence interval; MX, mammography; US, ultrasonography.

**Figure 10:**
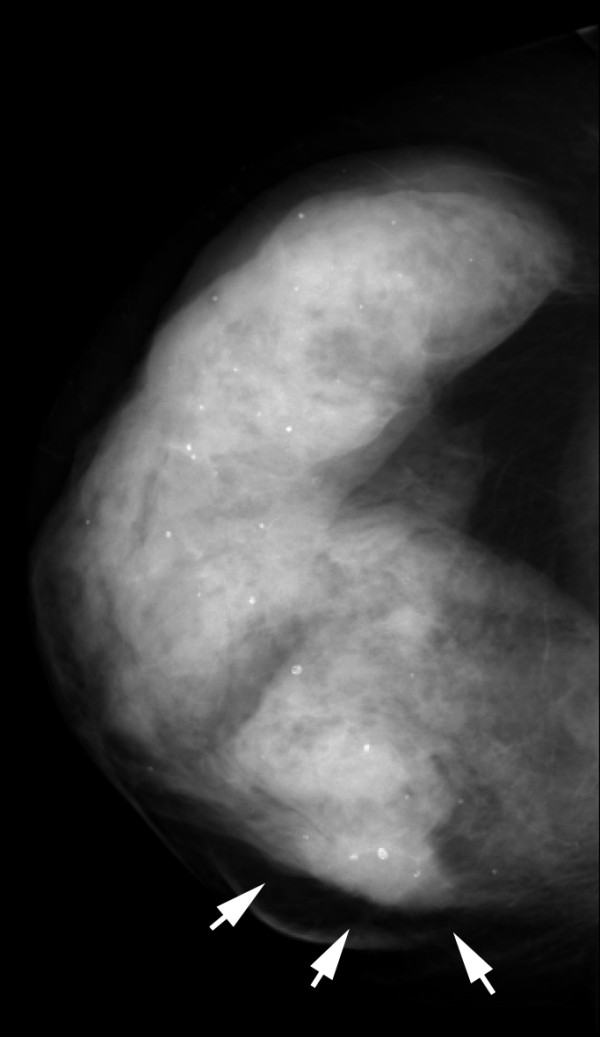
**Multifocal invasive ductal carcinoma in a 53-year-old woman with dense breasts**. The right craniocaudal view mammogram shows a very dense breast with an uncertain opacity in the inner quadrant (arrows).

**Figure 11:**
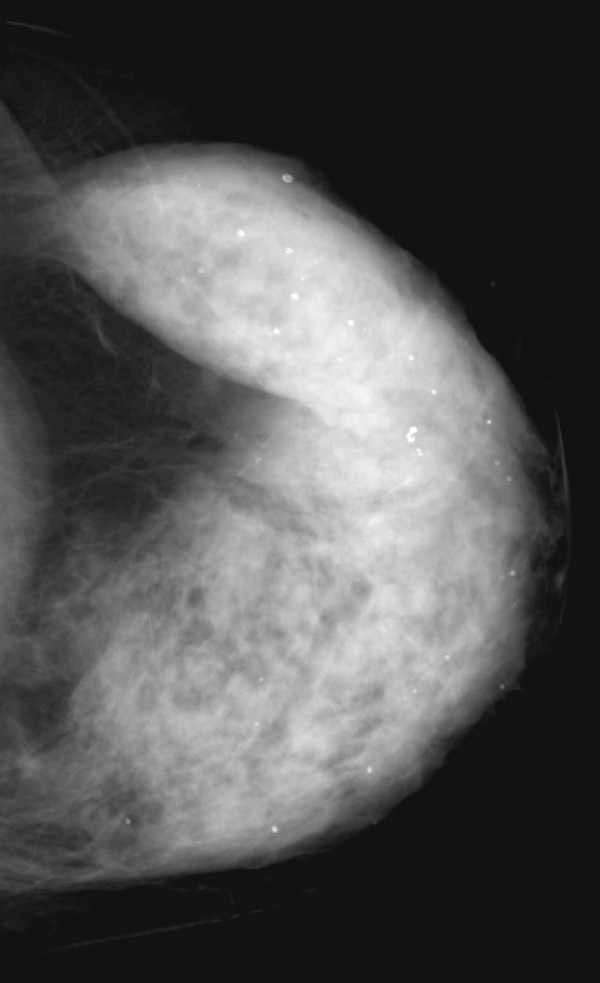
**Multifocal invasive ductal carcinoma in a 53-year-old woman with dense breasts**. The left craniocaudal view mammogram shows a very dense breast with no obvious lesion.

**Figure 12:**
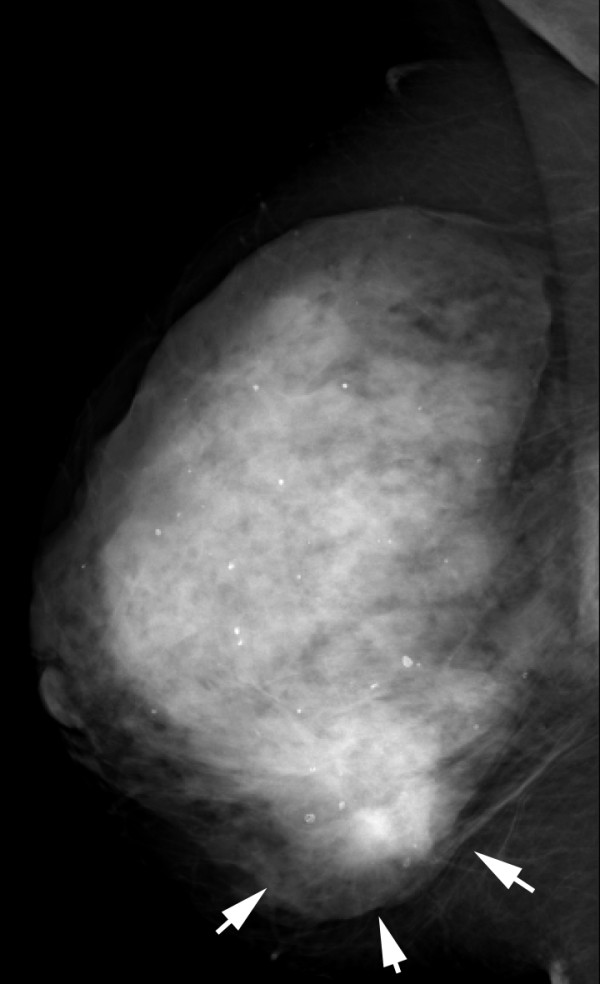
**Multifocal invasive ductal carcinoma in a 53-year-old woman with dense breasts**. The right mediolateral oblique view mammogram shows a very dense breast with an uncertain opacity in the inferior quadrant (arrows).

**Figure 13:**
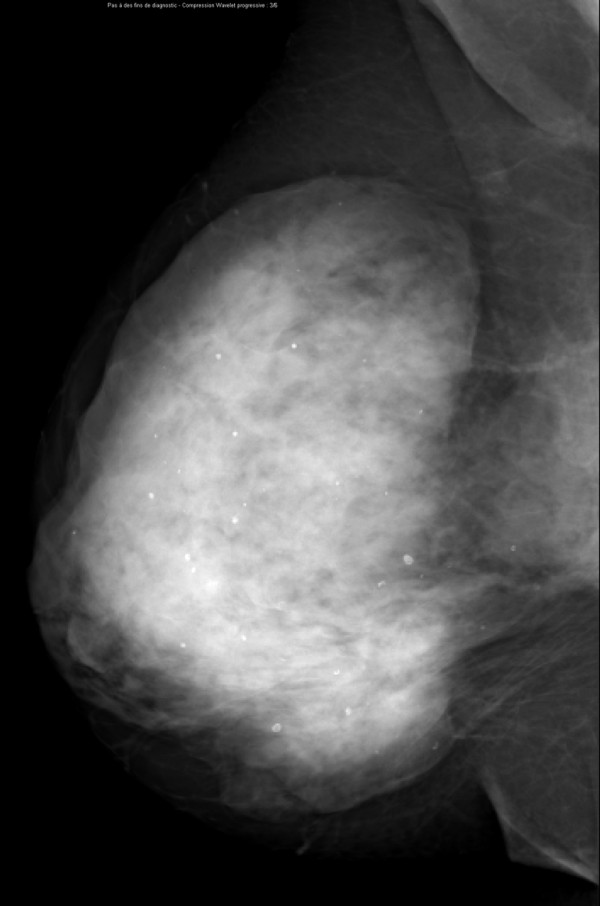
**Multifocal invasive ductal carcinoma in a 53-year-old woman with dense breasts**. The left mediolateral oblique view mammogram shows a very dense breast with no obvious lesion.

**Figure 14:**
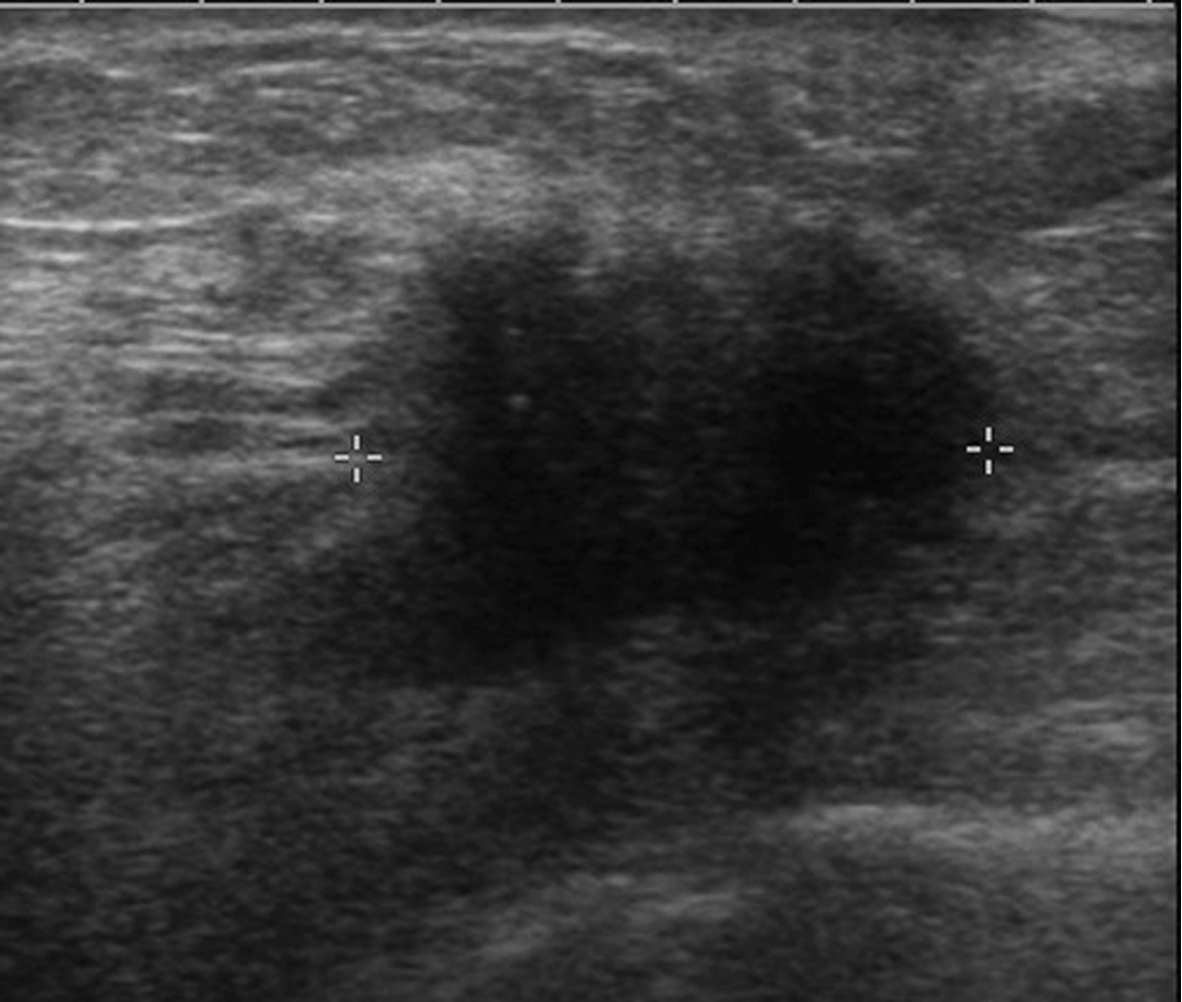
**Multifocal invasive ductal carcinoma in a 53-year-old woman with dense breasts**. The ultrasound image clearly demonstrates a 26-mm hypoechoic mass with irregular margins highly suggestive of malignancy.

**Figure 15:**
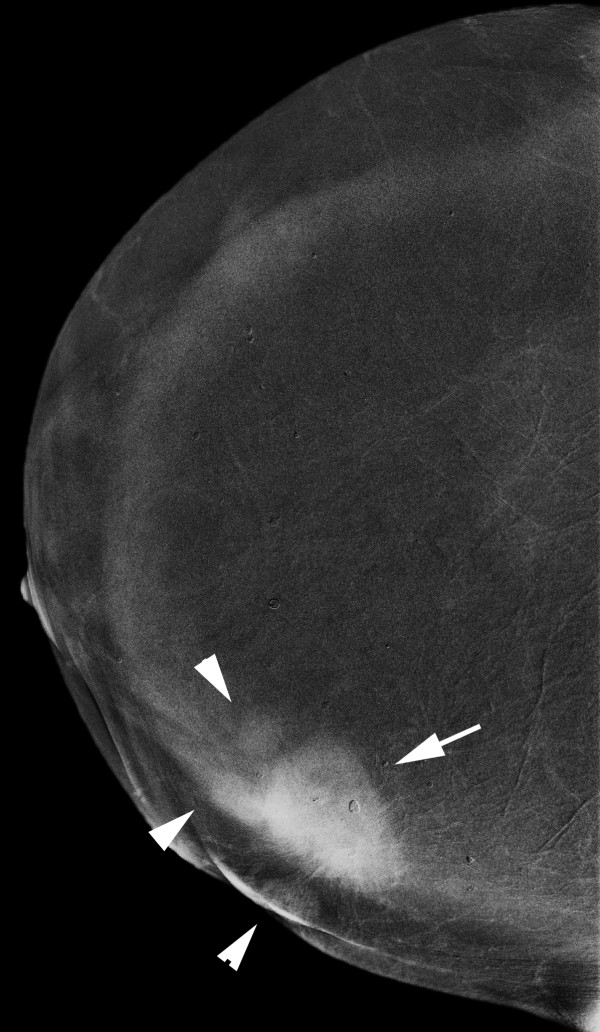
**Multifocal invasive ductal carcinoma in a 53-year-old woman with dense breasts**. The iodine-enhanced, contrast-enhanced digital mammography, right craniocaudal image readily depicts the main mass (arrow) and three additional adjacent nodules (arrowheads).

**Figure 16:**
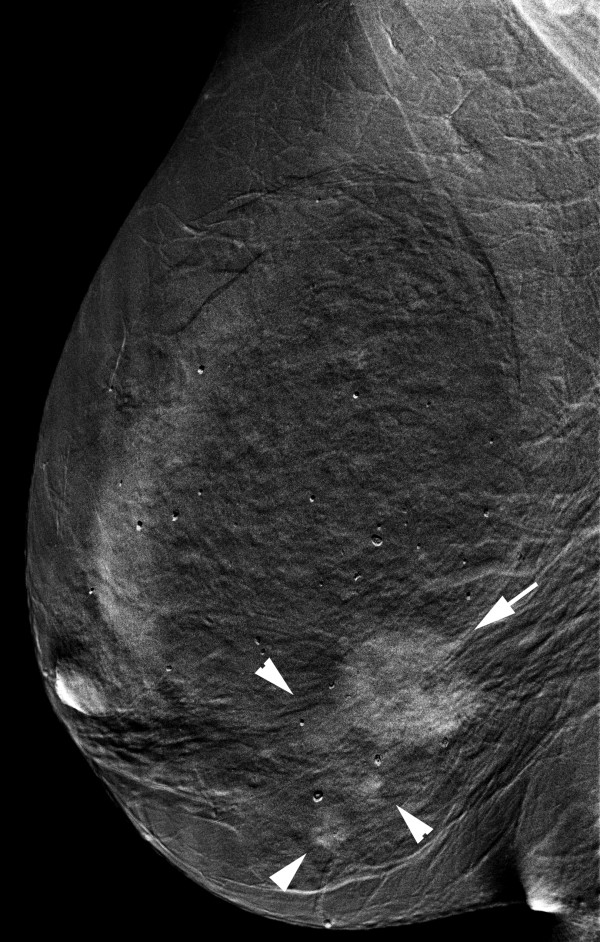
**Multifocal invasive ductal carcinoma in a 53-year-old woman with dense breasts**. The iodine-enhanced, contrast-enhanced digital mammography, right mediolateral oblique image readily depict the main mass (arrow) and three additional adjacent nodules (arrowheads).

**Figure 17:**
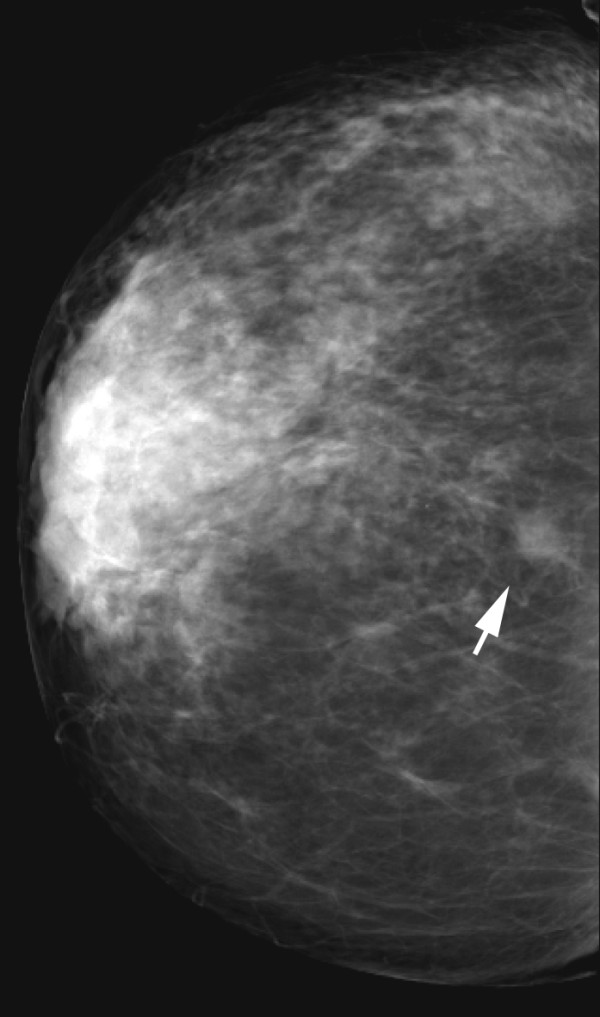
**Histologically proven normal breast parenchyma in a 69-year-old woman**. The right craniocaudal view mammogram shows an opacity with irregular margins (arrow).

**Figure 18:**
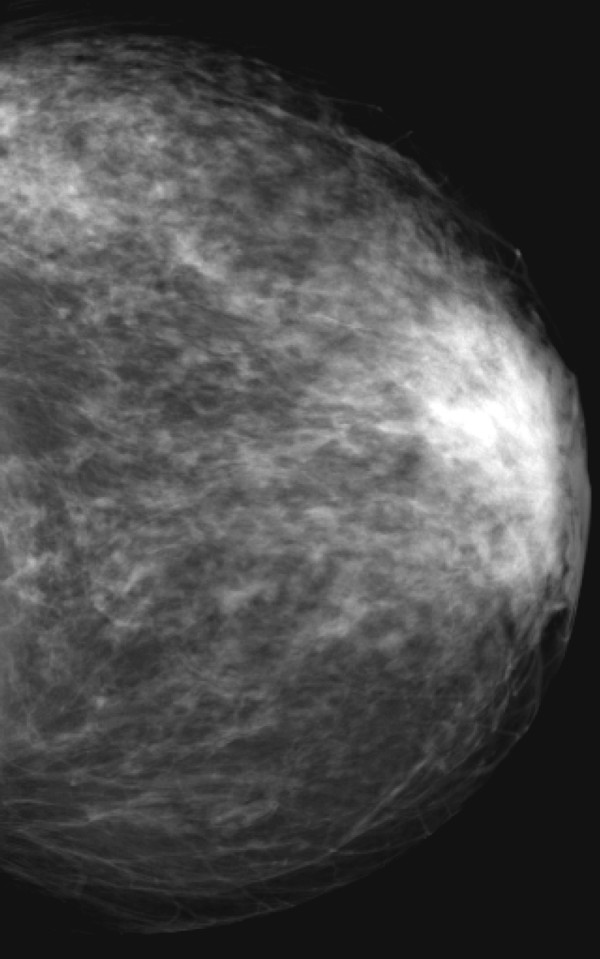
**Histologically proven normal breast parenchyma in a 69-year-old woman**. The left craniocaudal view mammogram is normal.

**Figure 19:**
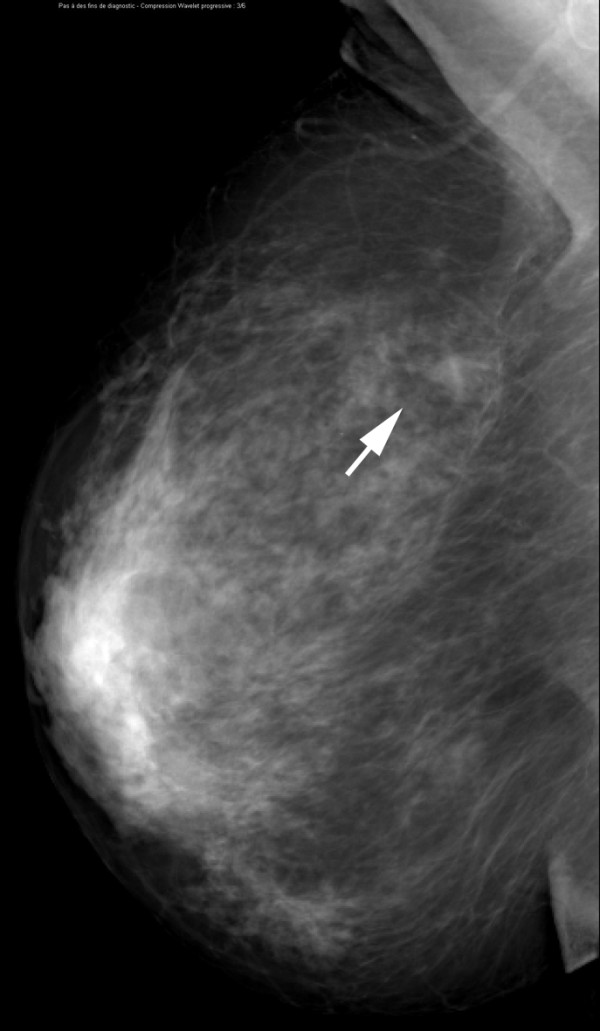
**Histologically proven normal breast parenchyma in a 69-year-old woman**. The right mediolateral oblique view mammogram shows an opacity with irregular margins (arrow). This lesion is classified as a BI-RADS (Breast Imaging, Reporting and Data System) score of 4.

**Figure 20:**
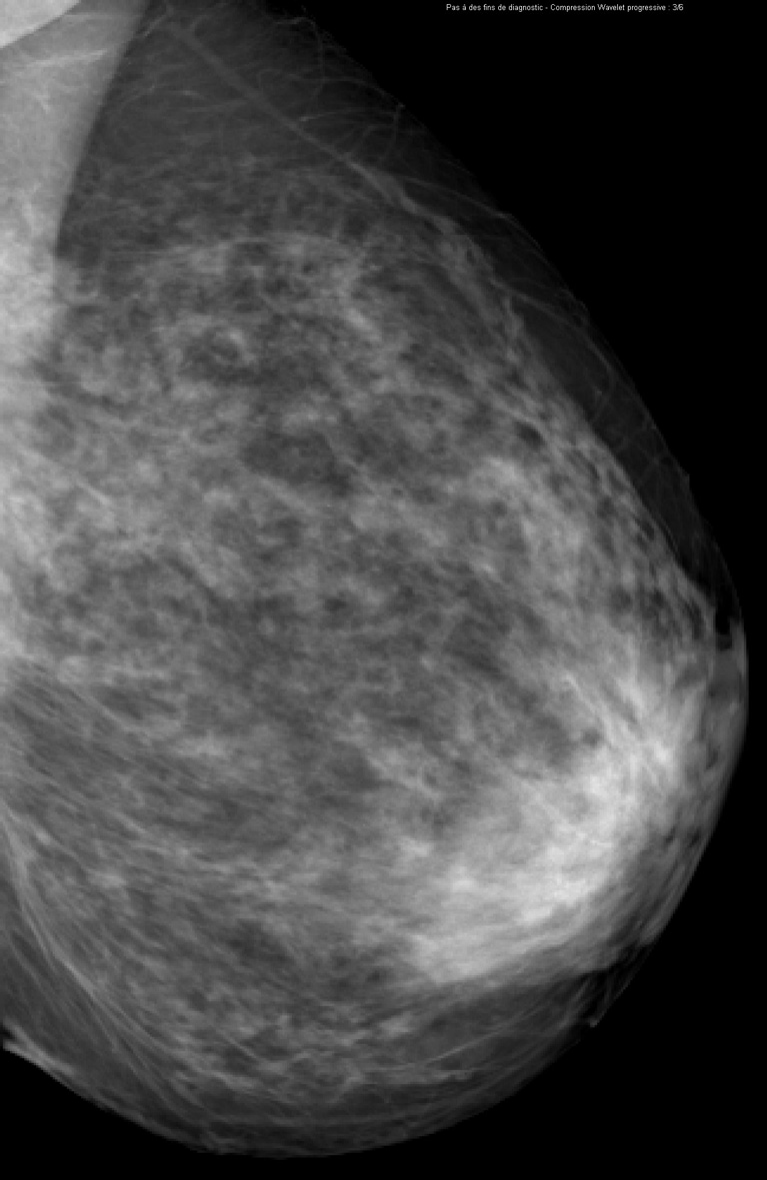
**Histologically proven normal breast parenchyma in a 69-year-old woman**. The left mediolateral oblique view mammogram is normal.

**Figure 21:**
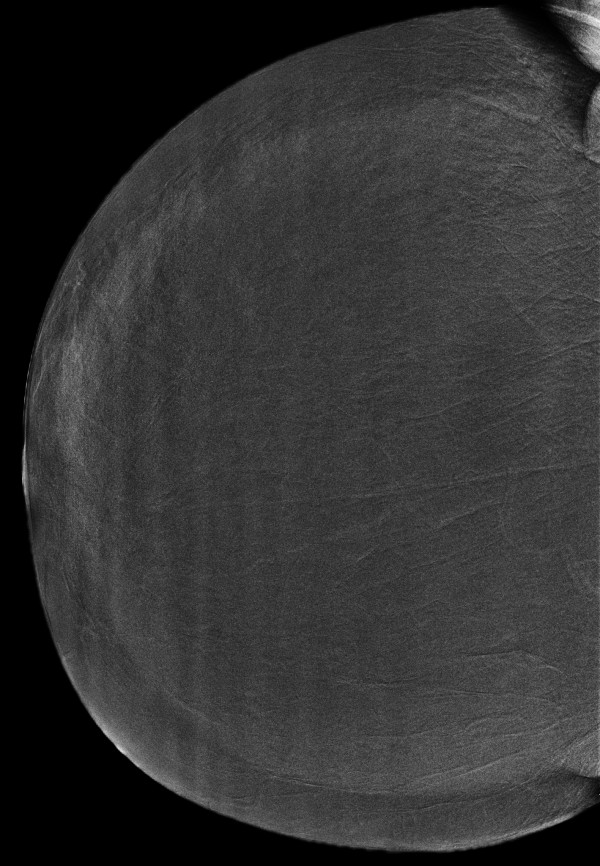
**Histologically proven normal breast parenchyma in a 69-year-old woman**. The iodine-enhanced, contrast-enhanced digital mammography, right craniocaudal image clearly demonstrates no obvious contrast uptake in the area of the nodule depicted on mammography.

**Figure 22:**
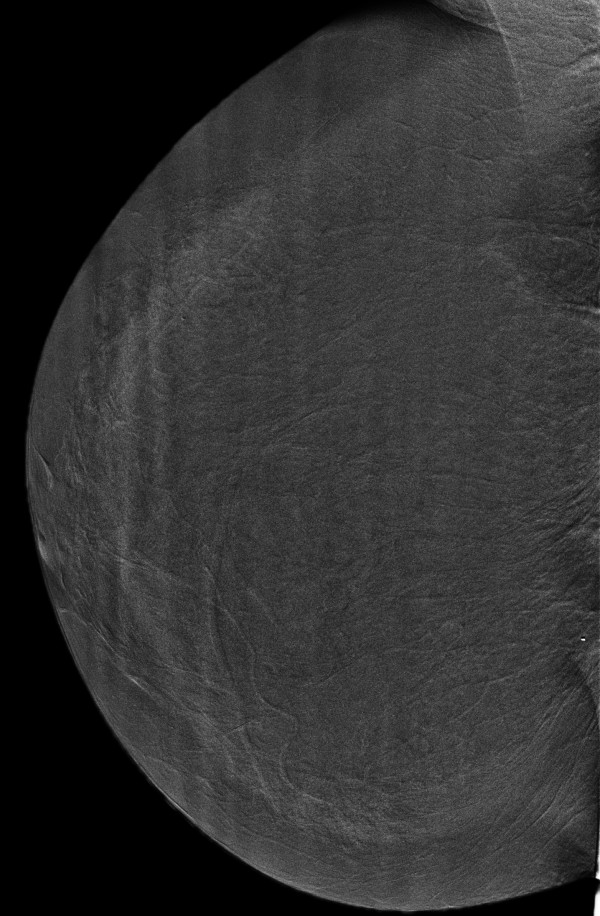
**Histologically proven normal breast parenchyma in a 69-year-old woman**. The iodine-enhanced, contrast-enhanced digital mammography, mediolateral oblique right image clearly demonstrates no obvious contrast uptake in the area of the nodule depicted on mammography. A core-needle stereotactic biopsy confirmed that the opacity was normal glandular tissue.

The addition of CEDM to MX and US enabled radiologists to find additional malignant lesions (Table 4) (Figures 10, 11, 12, 13, 14, 15, 16). Overall sensitivities were 0.712 (SE = 0.043) for MX ± US and 0.778 (SE = 0.040) for MX ± US ± CEDM. The increase in sensitivity of 0.065 (SE = 0.024) was statistically significant (*P *= 0.006). In patients without malignant lesions, the average number of reader findings with a BI-RADS score of 4 or 5 decreased for three of six study readers, did not change for one, and increased for two readers with the addition of CEDM (Figures 17, 18, 19, 20, 21, 22). Overall, there was a slight decrease in false-positive marks per case with the addition of CEDM, from 0.656 using MX ± US to 0.638 using MX ± US ± CEDM; this decrease was not statistically significant (-0.018 ± 0.050, *P *= 0.726 NS). Similar results were obtained by using the 7-point probability of malignancy scale.

**Table 4:** Per-lesion sensitivity to malignant lesions and false-positive marks per case using BI-RADS score of at least 4 to define a positive mark

Reader	MX ± US	MX ± US ± CEDM	Difference	*P *value
Sensitivity				
1	0.702 ± 0.050	0.762 ± 0.047	0.060	0.13
2	0.738 ± 0.048	0.81 ± 0.043	0.071	0.08
3	0.738 ± 0.048	0.845 ± 0.040	0.107	0.01
4	0.762 ± 0.047	0.821 ± 0.042	0.060	0.07
5	0.702 ± 0.05	0.738 ± 0.048	0.036	0.50
6	0.631 ± 0.053	0.690 ± 0.051	0.060	0.18
Overall	0.712 ± 0.043	0.778 ± 0.040	0.065 ± 0.024	0.006
95% CI	0.628 to 0.797	0.697 to 0.858	0.019 to 0.112	
False-positive marks per case (cases without malignant lesions)				
1	0.478 ± 0.056	0.652 ± 0.064	0.174	0.034
2	0.565 ± 0.056	0.435 ± 0.052	-0.130	0.128
3	0.652 ± 0.058	0.674 ± 0.067	0.022	0.745
4	1.043 ± 0.06	0.978 ± 0.065	-0.065	0.545
5	0.717 ± 0.052	0.609 ± 0.051	-0.108	0.134
6	0.478 ± 0.056	0.478 ± 0.063	0.000	1.000
Overall	0.656 ± 0.092	0.638 ± 0.089	-0.018 ± 0.050	0.726
95% CI	0.443 to 0.869	0.434 to 0.842	-0.135 to 0.099	

BI-RADS, Breast Imaging, Reporting and Data System; CEDM, contrast-enhanced digital mammography; CI, confidence interval; MX, mammography; US, ultrasonography.

Feature visibility for MX ± CEDM was rated as similar to MX ± US in 43.3% of study readings, slightly better in 21.2%, and better in 15.9% (Table 5). Overall, feature visibility on MX ± CEDM was rated similar to or better than feature visibility on MX ± US in 80.5% of ratings (95% CI 74.5% to 86.5%).

**Table 5:** Likert scale scores (*n *= 110 eligible patients)

Reader	MX ± US better	MX ± US slightly better	Similar	MX ± CEDM slightly better	MX ± CEDM better
1	6 (5)	10 (9)	39 (35)	24 (22)	31 (28)
2	11 (10)	15 (14)	51 (46)	16 (15)	17 (15)
3	3 (3)	20 (18)	56 (51)	25 (23)	6 (5)
4	5 (5)	10 (9)	47 (43)	26 (24)	22 (20)
5	19 (17)	10 (9)	45 (41)	25 (23)	11 (10)
6	8 (7)	12 (11)	48 (44)	24 (22)	18 (16)
Overall	8.7 (7.9)	12.8 (11.7)	47.7 (43.3)	23.3 (21.2)	17.5 (15.9)
Average MX ± CEDM similar, slightly better, or better				80.5%	
95% CI^a^				74.5% to 86.5%	

Values are presented as number (percentage by row). ^a^95% confidence interval (CI) obtained from a Student *t *distribution with 18.9 degrees of freedom (Hillis, 2007 [29]). BI-RADS, Breast Imaging, Reporting and Data System; CEDM, contrast-enhanced digital mammography; MX, mammography; US, ultrasonography.

## Discussion

Contrast-enhanced digital MX is a new breast imaging technique that aims at demonstrating breast carcinoma angiogenesis. Technical and clinical experience has been acquired and encouraging results have been published during the last few years on CEDM as an adjunct to MX [12-15]. Temporal subtraction was first tested with an approach similar to that of breast MRI [12-14]. These studies have shown the capability of CEDM to depict tumor angiogenesis in invasive breast cancer and have demonstrated contrast uptake in most malignant lesions. The main advantage of temporal subtraction is its ability to analyze the kinetics of time-enhancement curves. Kinetic curve assessment using CEDM, however, has failed to demonstrate clinical relevance [12-14]. Both benign and malignant breast tumors, evaluated by using a temporal CEDM technique, have shown progressive enhancement. Moreover, poor correlation was observed between the intra-tumoral mean vascular density evaluated on CD34-immunostained histological sections and quantitative characteristics of time-enhancement kinetics [14]. One hypothesis to explain this lack of washout in most cancers depicted with CEDM is that, unlike MRI, CEDM is a two-dimensional projection imaging technique and region-of-interest evaluations are made in a column of breast tissue that is the summation of enhancing tumor and enhancing surrounding normal breast parenchyma.

In this study, CEDM examinations were performed by using a dual-energy technique. Only one preliminary clinical study using the dual-energy technique has been published. Lewin and colleagues [11] examined 26 women (14 with malignant lesions and 12 with benign lesions) scheduled for breast biopsy with a pre- and post-contrast MLO acquisition. Twelve of the 13 invasive carcinomas (92%) demonstrated strong or moderate enhancement, and one demonstrated weak enhancement. Five of these invasive cancers were not detected on conventional MX. Of the 12 benign lesions, 10 (83%) demonstrated no enhancement and two demonstrated weak enhancement on CEDM images. The study examination by Lewin and colleagues included low- and high-energy exposures during a single breast compression in the MLO projection. The breast was then released from compression, and contrast agent was administered. After a delay of about 150 seconds, the breast was compressed again, and low- and high-energy exposures were repeated. From these images, pre- and post-contrast dual-energy images were created. High-energy images were acquired at 44 kVp with an 8-mm aluminum filter, and most low-energy images were acquired at 30 kVp with an Mo target-Mo filter combination or at 33 kVp with an Rh target-Rh filter combination.

The experimental setup that was used in our study has been significantly improved in comparison with the pioneering work of Lewin and colleagues. In our exams, the contrast agent was first administered to the patient two minutes before starting breast compression and image acquisition. Most low-energy images were acquired with an Rh target-Rh filter combination. Techniques for high-energy exposures were optimized to deliver the highest contrast-to-noise ratio for the lowest x-ray dose delivered to the patient [32]. Overall, those optimized parameters achieved similar contrast-to-noise ratios of iodine uptake in comparison with the acquisition parameters reported by Lewin and colleagues, but with a breast dose that is reduced by a factor of 2 (breast was 5 cm thick, 50% glandular). Moreover, optimized acquisition parameters yield high- and low-energy images that enable better texture cancellation when image recombination was applied [17].

More recently, we published the results of an initial single-reader evaluation of dual-energy CEDM in the same patient population as this study [15]. The purpose of that investigational study was to evaluate the clinical value of CEDM versus MX ± US. Encouraging results prompted the new study reported in this article, which involved six independent readers who evaluated CEDM, not compared with MX ± US but as an adjunct to MX ± US in diagnostic work-up, in order to reflect more accurately a potential routine clinical use of this technique. We performed a multireader review (six readers) of all images with per-reader and across-reader analyses. The addition of CEDM to MX ± US increased the ability of radiologists to discriminate between patients with malignant lesions and those without them. All individual readers improved their performance, and the area under ROC curves averaged across readers was significantly higher for MX ± US ± CEDM than for MX ± US. Moreover, the addition of CEDM to MX allowed the detection of more breast lesions with higher per-lesion sensitivity than MX ± US alone for all six readers, and the average breast cancer sensitivity based on BI-RADS score increased significantly from 71% to 78% without an increase in false positives. Indeed, CEDM imaging may help improve the visibility of suspicious findings and their differentiation thanks to its depiction of tumor angiogenesis. CEDM has the potential to increase breast cancer detection rates, improve staging of breast cancers, and improve patient selection for biopsy.

We chose to assess the adjunct of CEDM to MX ± US rather than MX alone because MX ± US corresponds to current clinical practice in the diagnostic setting. It could be useful, however, to perform CEDM before US because CEDM allows more accurate localization of the lesion than MX alone and provides better guidance for additional or second-look breast US. Moreover, in qualitative and subjective analysis using a Likert scale, CEDM was considered by radiologists to allow a clearer depiction of breast lesions. Indeed, the visibility of lesions with the addition of CEDM to MX was considered to be superior to MX ± US in more than 40% of cases, although some readers had little or no experience in interpreting CEDM images. These results highlight the contribution of contrast media injection in the depiction of breast cancers. CEDM adds functional information complementary to the morphologic findings of MX, aiding the detection and characterization of breast lesions.

The use of iodinated contrast agents, however, is not completely devoid of risk. Most adverse side effects are minor and have decreased considerably with the use of low-osmolality contrast media. Still, life-threatening reactions, though rare, can occur in the absence of any specific risk factors and with any type of contrast media [33]. All personnel (nurses, technologists, and radiologists) who administer contrast media must be fully prepared to treat even the most severe reaction, and adequate equipment and supplies must be available in the MX suite.

In our study, CEDM exams were performed on women recalled for work-up of findings unresolved after MX and US. CEDM was used as a problem-solving tool similarly to additional special MX views. Two-view (CC and MLO) dual-energy CEDM examinations were performed only on the suspicious breast. However, unlike the temporal subtraction technique, dual-energy CEDM has the potential to enable bilateral examinations with only one contrast agent injection and may be more convenient for staging newly diagnosed breast cancers. Dual-energy CEDM also allows shorter acquisition duration than temporal subtraction techniques and does not require extended breast compression. This could result in better acceptance from patients and fewer technical problems, such as misregistration of subtracted images. In dual-energy CEDM, contrast is injected without breast compression, thus avoiding patient motion linked to the heat sensation caused by the arrival of contrast agent and minimizing the impact of compression on contrast agent uptake in the breast.

## Conclusions

Dual-energy CEDM is a new and advanced clinical application of FFDM and is easily implemented, fast, and reproducible, and breast doses are comparable to those of standard digital MX. Dual-energy contrast-enhanced digital MX as an adjunct to MX ± US improves the diagnostic accuracy and the per-lesion sensitivity to malignant breast lesions of all readers in comparison with MX ± US alone and allows similar or improved visibility of breast lesions in most cases. CEDM may be a useful adjunct to diagnostic MX and a promising problem-solving and staging tool and may provide a cost-effective alternative to breast MRI for some clinical indications. Further research to evaluate the diagnostic accuracy and cost-effectiveness of CEDM compared with MRI is needed to define the appropriate role of CEDM in the future.

## Abbreviations

BI-RADS: Breast Imaging, Reporting and Data System; CC: craniocaudal; CEDM: contrast-enhanced digital mammography; CI: confidence interval; CT: computed tomography; FFDM: full-field digital mammography; kVp: peak kilovoltage; MLO: mediolateral oblique; Mo: molybdenum; MRI: magnetic resonance imaging; MX: mammography; NS: not significant; Rh: rhodium; ROC: receiver operating characteristic; SD: standard deviation; SE: standard error; US: ultrasonography.

## Competing interests

The authors declare that they have no competing interests.

## Authors' contributions

CD designed the study, recruited patients, acquired examinations, collected the data, carried out the analysis, and drafted the manuscript. FT and ATa participated in the design of the study and reviewed mammograms and CEDM examinations. FD, EMF, RJ, and MK reviewed mammograms and CEDM examinations. ATo participated in the design of the study and performed statistical analysis. REH helped to draft the manuscript. All authors read and approved the final manuscript.

## Authors' information

CD is a radiologist at the Institut de Cancérologie Gustave-Roussy (Villejuif, France). FT and ATa are radiologists at the Institut Curie (Paris, France). FD is a radiologist at the University Hospital Charite (Berlin, Germany). RJ is a radiologist at the Sunnybrook Health Sciences Center (Toronto, ON, Canada). MK is a radiologist at the University of North Carolina at Chapel Hill (Chapel Hill, NC, USA). REH is a radiologist at the University of Colorado-Denver, School of Medicine (Denver, CO, USA). ATo is an independent biostatistician at the Statistics Collaborative (Washington, DC, USA).
